# Laser speckle contrast imaging for blood flow monitoring in predicting outcomes after cerebral ischemia-reperfusion injury in mice

**DOI:** 10.1186/s12868-022-00769-x

**Published:** 2022-12-27

**Authors:** Lu Yin, Tengfei Yu, Linggang Cheng, Xinyao Liu, Wei Zhang, Hongxia Zhang, Lijuan Du, Wen He

**Affiliations:** Department of Ultrasound, Beijing Tiantan Hospital, Capital Medical University, Beijing, 100070 China

**Keywords:** Cerebral ischemia-reperfusion injury, Laser speckle contrast imaging, Cerebral blood flow, Brain tissue injury, Outcome prediction

## Abstract

**Background:**

In the treatment of ischemic cerebral stroke (ICS), most conventional treatments, including carotid endarterectomy and carotid artery stenting, may cause cerebral ischemia-reperfusion injury (CIRI). For treated ICS patients, changes in cerebral blood flow are directly related to brain function. At present, computed tomography perfusion, dynamic susceptibility contrast-enhanced perfusion weighted imaging and magnetic resonance arterial spin labeling perfusion imaging are used to monitor cerebral blood flow, but they still have some limitations. Our study aimed to monitor the changes in cerebral cortical blood flow by laser speckle contrast imaging (LSCI) in CIRI model mice and to propose a new method for predicting outcomes after CIRI. C57BL/6 N mice were used to establish a mouse CIRI model based on a modified thread-occlusion method and divided into a good outcome group and a poor outcome group according to survival within 7 days. The cerebral cortical blood flow of the area supplied by the left middle cerebral artery was monitored by LSCI at baseline (before modeling), 1 h after ischemia, immediately after reperfusion and 24 h after reperfusion. Then, the brains of the mice were removed immediately and stained with hematoxylin and eosin to observe the pathological changes in brain neurons.

**Results:**

The cerebral cortical blood flow in the poor outcome group was obviously reduced compared with that less in the good outcome group at 24 h after reperfusion (180.8 ± 20.9 vs. 113.9 ± 6.4, p = 0.001), and at 24 h after reperfusion, the cerebral cortical blood flow was negatively correlated with the severity of brain tissue injury (p = − 0.710, p = 0.010).

**Conclusions:**

LSCI can monitor the changes in cerebral cortical blood flow during CIRI in mice and could be used as a feasible method for predicting outcomes after CIRI in mice.

## Background

With the aging of society, stroke has become one of the main diseases that restrict the development of social health and the economy. The number of stroke patients increases by more than 1 million every year in China and ischemic cerebral stroke (ICS) accounts for approximately 80% [1]. In the treatment of ischemic cerebrovascular disease, most conventional treatments, including carotid endarterectomy (CEA) and carotid artery stenting (CAS), may cause cerebral ischemia-reperfusion injury (CIRI). The pathological process of CIRI includes the inflammatory response, oxidative stress, free radical production, brain edema and neuronal apoptosis or death, which are all induced by ICS [2]. The blood flow in the brain tissue changes from low perfusion to high perfusion, causing brain damage and leading to headaches, high blood pressure, cognitive impairment, focal neurological deficits, seizures, brain edema, intracranial hemorrhage or even death, and these clinical symptoms are known as cerebral hyperperfusion syndrome (CHS) [3]. CHS caused by CIRI seriously threatens human health. The incidence of CHS after CEA and CAS was 0.3–1.2% and 1.1–6.8%, respectively, and the mortality rate without intervention was as high as 50% [4, 5]. Therefore, it is quite important to achieve early recognition of CIRI and CHS.

For treated ICS patients, changes in cerebral blood flow are directly related to brain function. Understanding brain microcirculation changes is highly important for CIRI treatment and rehabilitation, and monitoring the parameters of brain blood dynamics can help clinicians to better understand the pathological progress of CIRI and guide treatment and rehabilitation. Compared with the complex indexes of cerebral hemodynamics and rheology, cerebral blood flow changes are more intuitive during CIRI. Therefore, the measurement of cerebral blood flow can be used as a direct reference for cerebral hemodynamic changes.

At present, computed tomography perfusion [6], dynamic susceptibility contrast-enhanced perfusion weighted imaging [7] and magnetic resonance arterial spin labeling perfusion imaging [8] are commonly used to evaluate cerebral blood perfusion. Although the above evaluation methods can fully reflect cerebral blood flow and the opening of the collateral circulation, the invasion, radiation risk, artifacts, and contraindications to examination still limit their clinical applications.

Laser speckle contrast imaging (LSCI) is a wide-field, noninvasive imaging technique with high temporal and spatial resolution, that is based on the scattering properties of moving particles (e.g., red blood cells) [9]. LSCI obtains the velocity information of the scattering particles and has been successfully used for neurovascular imaging. Biose et al. observed changes in cerebral collateral perfusion in an arterial occlusion rat model by using LSCI and found that hyperglycemia or preexisting hypertension impaired the dynamic blood supply of the cortical collaterals after arterial occlusion and aggravated ischemic stroke [10]. At present, most studies have focused on ICS, but relatively few studies have focused on cerebral cortical blood flow changes in CIRI.

The aim of this study was to observe the changes in cerebral cortical blood flow monitored by LSCI for predicting outcomes after CIRI in mice.

## Results

### Grouping

A total of 18 mice were used in this study, of which 5 mice were designated as the sham group. The remaining 13 mice were suffered from the CIRI modeling, among which 1 mouse died due to cerebral hemorrhage during the operation and 2 mice were excluded for not meeting the 5-point Longa scoring criteria [11]. Finally, 10 mice were included. Seven mice that survived for more than 7 days were classified into good outcome group, while 3 mice that died within 7 days were classified into poor outcome group.

### Comparison of cerebral cortical blood flow monitoring by LSCI at different time points

**Fig. 1:**
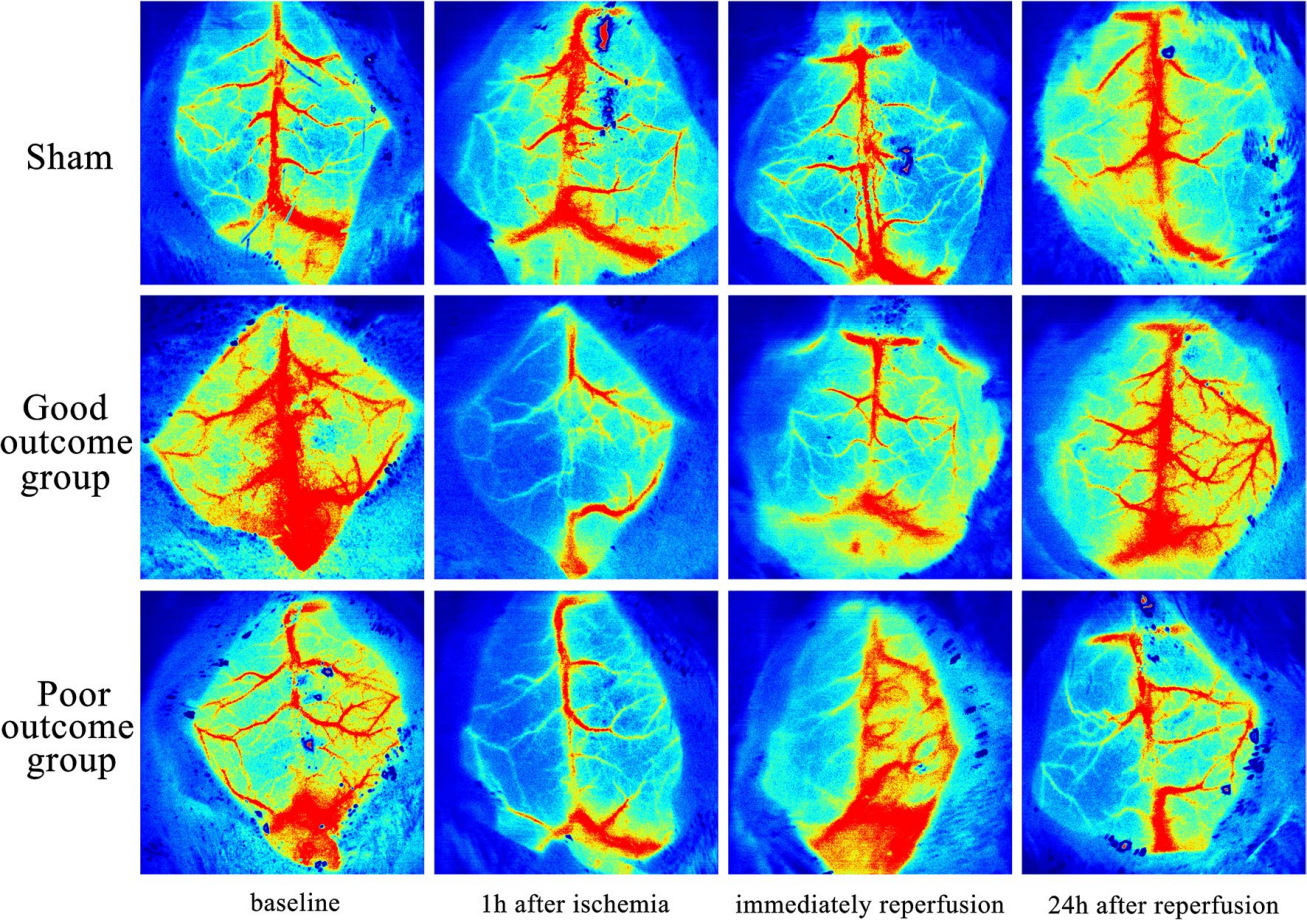
Representative LSCI imaging of cerebral cortical blood flow monitoring at different time points in the (**a**) sham group, (**b**) good outcome group and (**c**) poor outcome group. The blood flow in the area supplied by the left MCA in the good outcome group was greater than that in the poor outcome group at 24 h after reperfusion. *LSCI* laser speckle contrast imaging, *MCA* middle cerebral artery

Before modeling, there were no significant differences in cerebral cortical blood flow in the ROI of the left cerebral cortex area among sham group, good outcome group and poor outcome group in the left side (244.5 ± 60.5 a.u. vs, 256.4 ± 42.1 a.u. vs. 251.5 ± 28.8 a.u., p = 0.913). The detailed data were shown in Table 1. After ischemia, cerebral cortical blood flow was interrupted and decreased in the area supplied by the left MCA. At the initial stage of reperfusion, the cerebral cortical blood flow in both the good outcome group and poor outcome group recovered but was still lower than that before ischemia(117.5 ± 14.8 a.u. vs. 256.4 ± 42.1 a.u., P < 0.001 and 105.2 ± 8.4 a.u. vs. 251.5 ± 28.8 a.u., P = 0.007, respectively). At 24 h after reperfusion, the blood flow in both the good outcome group and the poor outcome group was still lower than that before ischemia; however, the blood flow in the good outcome group was greater than that in the poor outcome group and the difference was statistically significant(180.8 ± 20.9 vs. 113.9 ± 6.4, p = 0.001) (Fig. 1). For the right side, compared with sham group, there was a decline in the value of the right cerebral blood flow at 1 h after ischemia, immediately reperfusion and 24 h after reperfusion. However, there was no statistically significant difference among groups (all p > 0.05, Table 2).

**Table 1:** The cerebral cortical blood flow calculated by LSCI in the left cerebral cortex area

Groups	Values in ROI (a.u.)			
	Baseline	1 h after ischemia	Immediately reperfusion	24 h after reperfusion
Sham n = 5	244.5 ± 60.5	244.1 ± 47.5	239.0 ± 52.9	241.8 ± 50.8
Good outcome n = 7	256.4 ± 42.1	63.9 ± 14.1^a^	117.5 ± 14.8^a^	180.8 ± 20.9^a^
Poor outcome n = 3	251.5 ± 28.8	63.4 ± 9.6^a^	105.2 ± 8.4^a^	113.9 ± 6.4^a, b^

a: p < 0.05, compared with the sham group; b: p < 0.05, compared with the good outcome group.

*LSCI* laser speckle contrast imaging

**Table 2:** The cerebral cortical blood flow calculated by LSCI in the right cerebral cortex area

Groups	Values in ROI (a.u.)			
	Baseline	1 h after ischemia	Immediately reperfusion	24 h after reperfusion
Sham n = 5	248.5 ± 56.2	246.2 ± 57.8	239.3 ± 45.4	239.5 ± 45.7
Good outcome n = 7	243.7 ± 47.1	198.3 ± 38.7	211.5 ± 40.5	226.6 ± 44.9
Poor outcome n = 3	244.3 ± 40.9	166.8 ± 32.1	194.0 ± 34.9	215.0 ± 42.2

*LSCI* laser speckle contrast imaging.

All p > 0.05 between good outcome group and poor outcome group

### Hematoxylin and eosin (HE) staining

**Fig. 2:**
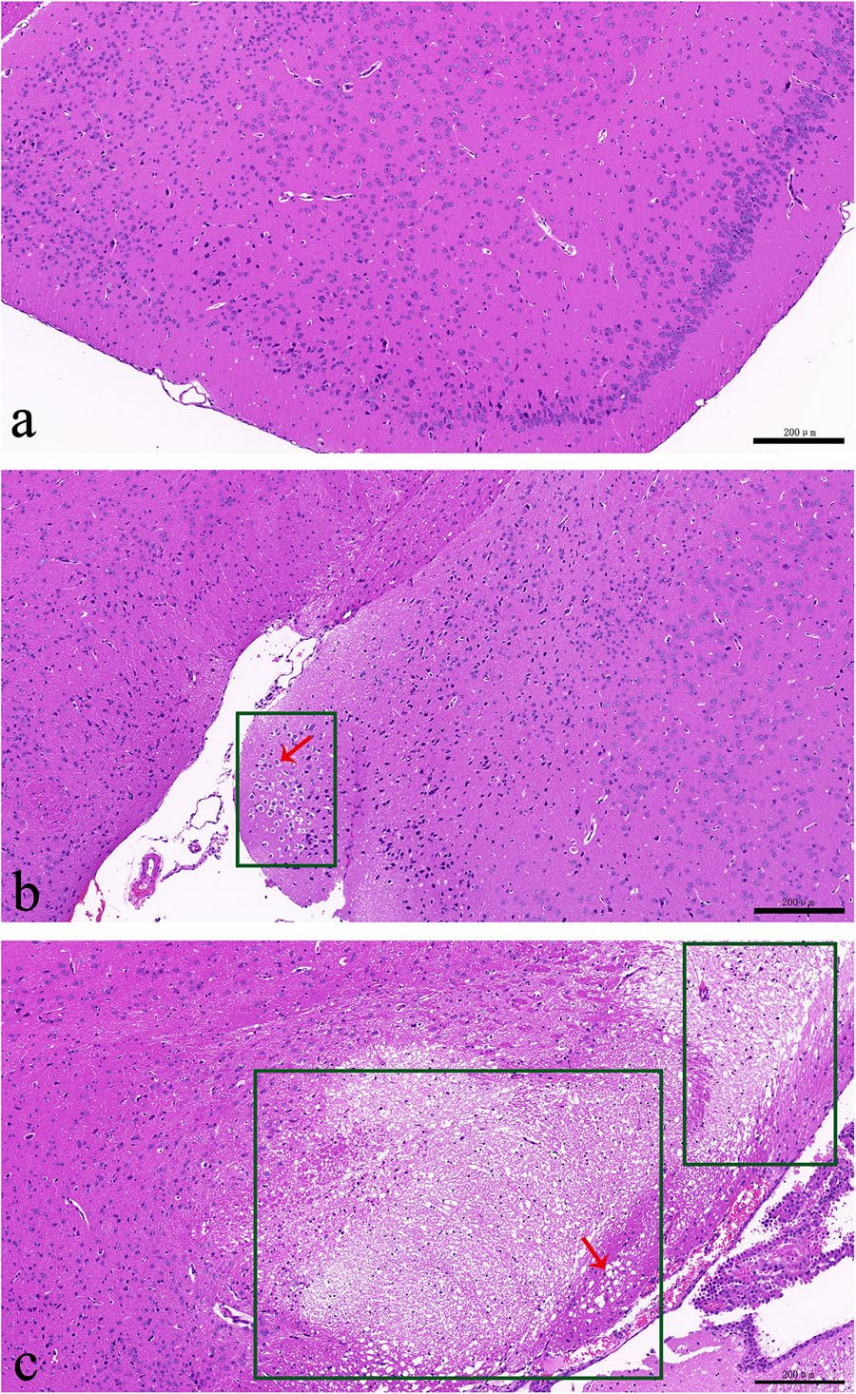
HE staining of brain tissues. **a** Normal tissue: intact nuclei of brain neurons with clear cell membrane; **b** mild injury: cell swelling with disordered arrangement and interstitial edema; **c** severe injury: a large number of necrotic cells and severe damage to the tissue structure. Green boxes indicated the damaged areas, and red arrows indicated swollen and necrotic cells. *HE* Hematoxylin and eosin. (Magnifcation: 400x; measured resolution at acquisition: 96 dpi; enhanced resolution for publication by Adobe Photoshop CS6 version 13.0: 300 dpi)

We classified the degree of brain tissue injury into normal, mild and severe according to the HE staining results. Microscopic results showed that the nuclei of brain neurons in the sham group were clear and intact, and the cell membrane was clear without tissue edema. The brain neurons with mild injury showed swelling with a disordered arrangement and interstitial edema. In severe brain tissue injury, necrosis of brain neurons in the poor outcome group was aggravated, with a large number of necrotic cells and severe damage to the tissue structure (Fig. 2).

### Relationships between cerebral cortical blood flow monitored by LSCI and the degree of brain tissue injury

**Fig. 3:**
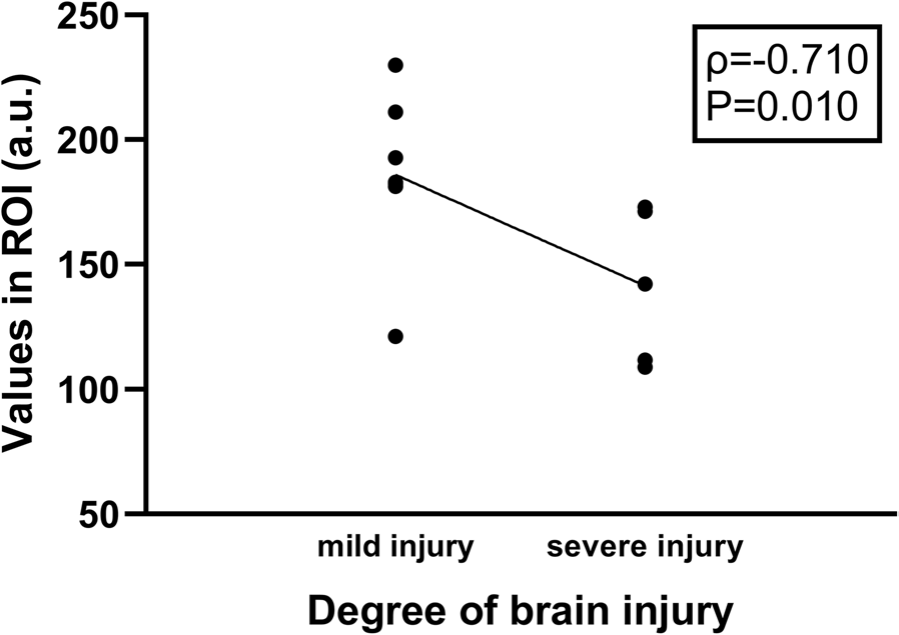
Relationships between cerebral cortical blood flow monitored by LSCI and the degree of brain tissue injury. The lower the cerebral blood flow value was, the more severe the degree of brain tissue injury was. *LSCI* laser speckle contrast imaging

We found that there was a negative correlation between cerebral cortical blood flow at 24 h after reperfusion and the degree of brain tissue injury(p = − 0.710, P = 0.010). The lower the cerebral blood flow value was, the more severe the degree of brain tissue injury was (Fig. 3).

## Discussion

The changes in cerebral cortical blood flow were monitored by using LSCI before monitoring for ischemia to 24 h after reperfusion in mice at different time points in this study. The results showed that cerebral cortical blood flow in mice decreased obviously in the ischemia period and rose immediately after reperfusion, but it was still less than that before ischemia. At 24 h after reperfusion, the cerebral cortical blood flow in the poor outcome group was obviously less than that in the good outcome group. Moreover, the value of cerebral cortical blood flow at 24 h after reperfusion was negatively correlated with the severity of brain tissue injury.

LSCI, which can image the velocity distribution of blood flow in real time with high temporal and spatial resolution, has incomparable advantages [12]. In this study, the changes in cerebral cortical blood flow during CIRI in mice were observed by LSCI, which verified the convenience and feasibility of LSCI for monitoring cerebral blood flow in real time and throughout the whole process [13]. Although LSCI cannot directly measure absolute velocity at present, the experimental equipment is simple to use, inexpensive, noninvasive, and fast and captures real-time data to obtain the two-dimensional flow velocity distribution map, which is clear and intuitive for scientific researchers or clinicians. Therefore, there are still certain developments and potential applications in blood flow detection and other biomedical fields.

Cerebral blood flow plays a very important role in maintaining normal physiological function in human beings. The monitoring of cerebral blood flow is conducive not only to exploring the pathophysiological changes in blood flow in local lesions in ICS and other brain diseases [14, 15] but also to improving the efficiency of clinical prevention and treatment. In this study, we found that the cerebral cortical blood flow in the poor outcome group was obviously less than that in the good outcome group at 24 h after reperfusion and that the value of cerebral cortical blood flow at 24 h after reperfusion was negatively correlated with the severity of brain tissue injury. This may be because of the more severe inflammatory response, oxidative stress, free radical production, brain edema and neuronal apoptosis or death leading to an obstruction of blood reperfusion and collateral circulation. The reverse is also possible, and low blood flow leads to more severe brain tissue damage. The specific mechanism needs to be further studied.

Our study also had some limitations. (1). Only the changes in cerebral blood flow in the area of the cerebral cortex supplied by the MCA were studied, but the changes in blood flow in the MCA itself were not studied; and (2) cerebral cortical blood flow was observed only within 24 h after CIRI, and observation at other time points may also be needed. Despite the above deficiencies, we proposed a new method for predicting the prognosis of CIRI by using LSCI to monitor the changes in cerebral cortical blood flow during CIRI in mice in this study and the results indicated that LSCI could be used to predict outcomes after CIRI in mice.

## Conclusions

LSCI could monitor the changes in cerebral cortical blood flow during CIRI in mice and could be used as a feasible method to predict outcomes after CIRI in mice.

## Methods

All animal studies were approved by the Ethic Committee of Beijing Tiantan Hospital.

### Main materials and instruments

C57BL/6 N mice (male, 7–8 weeks, 23 ± 2 g) were obtained from Charles River (Beijing, China). The mice had free access to food and water and were housed at 25 ± 1 °C under a 12 h light/12 h dark cycle. A laser speckle imaging system ((RFLSI III, RWD Life Science, China) and sutures for mice weighing 20–25 g (RWD Life Science, China) were used.

### Establishment of the mouse CIRI model

Before the experiment, the mice were fasted for 12 h and provided free access to water. The CIRI mouse model was induced by middle cerebral artery occlusion (MCAO) according to a previous study [16]. Briefly, the mice were intraperitoneally anesthetized with 2% pentobarbital sodium (45 mg/kg). A silicon-coated suture was then inserted from the distal left common carotid artery and advanced into the internal carotid artery to reach and obstruct the middle cerebral artery (MCA) for 1 h, and then the silicon-coated suture was removed out for reperfusion.

The neurological function of the mice was scored according to the 5-point Longa scoring method [11], and the scoring criteria were as follows: a score of 0 means no neurologic deficit, a score of 1 means failure to extend the contralateral forepaw fully, a score of 2 means circling to the contralateral side, a score of 3 means falling to the contralateral side and a score of 4 means not walking spontaneously and having a depressed level of consciousness. Mice with a score of 2–3 were included in the subsequent experiment.

### Grouping

The mice that met the scoring criteria were divided into a good outcome group and a poor outcome group according to observations within 7 days. Mice that died within 7 days were considered to have a poor outcome and mice that survived more than 7 days were considered to have a good outcome. In addition, five mice underwent only incision of the neck skin and vascular separation and were designated as the sham group.

### Cerebral cortical blood flow monitoring by LSCI

After anesthesia, the mice were randomly fixed in the prone position on a thermostatic mouse plate to receive the cerebral cortical blood flow monitoring on both sides by LSCI. The operators were blinded to the group allocation. The skin and mucous membrane of the head were separated to keep the skull fully exposed. The LSCI system was focused on the mouse skull to obtain a clear color map. Normal saline was instilled on the skull to maintain a wet condition. The cortical blood flow of the both cerebral hemispheres supplied by the MCA was monitored at baseline (before modeling), 1 h after ischemia, immediately after reperfusion and 24 h after reperfusion. Keeping the mice under anesthesia to obtain stable breathing and heart rates was necessary during the whole monitoring process. Continuous monitoring was performed for 15–30 s. The region of interest (ROI) was set, and the value of the ROI represents the blood flow in the selected region.

### HE staining

Mice were anesthetized by 1% isoflurane (R510-22, RWD, China) using a gas anesthesia machine (R540IP, RWD, China) and subsequently perfused by injecting 4% paraformaldehyde (60 ml) through the heart. The brain was removed and fixed with 4% paraformaldehyde. The left damaged brain tissue was dissected to embed in paraffin and then sectioned using a microtome (RM2016, Leica, Germany) with a thickness of 5 μm. The sections were baked in an oven at 60 °C and dewaxed, and then stained with Harris hematoxylin for 3–8 min. After this, the sections were stained with eosin for 1–3 min after washing twice with tap water. Finally, the sections were dehydrated twice with 95% alcohol (5 min each) and washed three times with xylene (5 min each), and then fixed with natural gum. All sections were scanned with a digital pathology scanner system (Pannoramic 250, 3D HISTECH Ltd., Hungary), and 5 fields in injured areas were randomly selected for analysis. The output digital pathological images were 400x enlarged.

### Statistical analysis

SPSS version 21.0 (IBM Corp., Armonk, NY, US) was used for statistical analysis, and the data are expressed as the mean ± standard deviation. One-way ANOVA was used to compare differences between groups, and p < 0.05 indicated that the difference was statistically significant. The formal sample size has not been decided due to insufficient data. However, based on previous studies [17–19], 3–5 mice per group could meet the statistical requirements.

## Data Availability

All data generated or analyzed during this study are included in this published article.

## Abbreviations

CAS: Carotid artery stenting
CEA: Carotid endarterectomy
CHS: Cerebral hyperperfusion syndrome
CIRI: Cerebral ischemia-reperfusion injury
HE: Hematoxylin and eosin
ICS: Ischemic cerebral stroke
LSCI: Laser speckle contrast imaging
